# Impact of intense disturbance on the structure and composition of wet-eucalypt forests: A case study from the Tasmanian 2016 wildfires

**DOI:** 10.1371/journal.pone.0200905

**Published:** 2018-07-20

**Authors:** Tamika J. Lunn, Melissa Gerwin, Jessie C. Buettel, Barry W. Brook

**Affiliations:** School of Natural Sciences, ARC Centre of Excellence for Australian Biodiversity and Heritage, University of Tasmania, Sandy Bay, Tasmania, Australia; Chinese Academy of Forestry, CHINA

## Abstract

Fire is a key process in eucalypt communities, exerting a strong influence on the composition, structure and functioning of forests. Much of the research on the fire response of temperate, wet-sclerophyll trees in Australia comes from Victoria, where the dominant eucalypt is *Eucalyptus regnans*. In contrast, central and northern Tasmanian forests, dominated by *Eucalyptus delegatensis*, are relatively understudied. There is a need to determine whether Tasmanian wet-sclerophyll forests, though the same forest type in name, are functionally different in floristics and response to fire. Here we document the forest community response to a natural wildfire event in Tasmania—using opportunistic before/after control/impact (BACI) data from pre-existing monitoring plots. Uniting pre- and post-fire floristic data, we quantified mortality and regeneration of eucalypt, acacia and other dominant tree species, and tree ferns, *Dicksonia antarctica*, in response to wildfire. We also evaluated the density of eucalypt and acacia seedling establishment between burnt and unburnt forests, and quantified faunal responses to fire. Despite moderate-to-high intensity burning in patches across the plot, mortality of eucalypts, acacias and tree ferns due to fire were low. By contrast, fire-sensitive rainforest species showed low survival, though were able to persist in unburnt refugia. Eucalypt and acacia seedling regeneration was high in the burnt plot, suggesting that *E*. *delegatensis* forests regenerate without stand-replacing fire events. This contrasts to Victorian *E*. *regnans* forests, whose persistence is dependent on high-severity stand-replacing events. We also found some group-specific avifaunal and invertebrate responses to the fire event, which are broadly reflective of responses documented in other Victorian-based studies. Our results have implications for Tasmanian wet-forest silvicultural practices, which are based on the principle of stand-replacement after fire. The broader relevance of this work to forest ecology is in demonstrating the serendipitous opportunities that can arise with baseline monitoring plots.

## Introduction

Disturbances are important natural drivers of forest-ecosystem dynamics, and strongly influence the composition, structure and functioning of their communities [1]. Forest-disturbance regimes are intensifying in many parts of the world, with climate change expected to drive further changes in the size, severity and frequency of disturbance events globally [2, 3]. Wildfire in particular is an important and climate-sensitive disturbance agent which can cause severe impacts on forests [3]. Some effects are direct (e.g., tree mortality), while others persist across multiple temporal and spatial scales by creating an adaptive advantage for fire resilience and shaping species patterns and occurrence across the landscape [4].

Future changes to disturbance regimes are likely to alter forest ecosystems across the globe, with potential far reaching impacts on their biological diversity. A recent example of forest disturbance, driven at least in part by climate change, was the Jan/Feb 2016 Tasmanian wildfires (southern Australia), which burnt 97,000 hectares of forest across the state [5]. These fires were attributed to a record-breaking dry spring, followed by a continued dry and warm summer, which left long-unburnt areas uncharacteristically dry and vulnerable to ignition by lightning. Within this devastated landscape, relict alpine species, which had existed in a landscape without fire for centuries, were severely burned, and large patches of the community were lost. With the occurrence of such fire events predicted to increase in severity and frequency into the future, the risk of losing species and landscapes that are maladapted to fire will continue to increase. Even forest systems that are well adapted to fire are vulnerable to population collapse, if fire frequencies increase as abruptly as anticipated [6]. Understanding how individual species respond to fire events will be critical for preserving biodiversity and ecosystem structure in the face of climate change.

Tall eucalypt forests in Australia are strongly fire-driven ecosystems, requiring a specific fire regime for their continued existence [7]. Much of the research on the fire response of temperate wet-sclerophyll forest has been done in Victorian forests (south-eastern part of continental Australia), where the dominant eucalypt is *Eucalyptus regnans* [8, 9]. This species is an obligate seeder, regenerating exclusively from seed after fire, and is one of the fastest growing tree species in the world. Although it is readily killed by crown fire, *E*. *regnans* is also highly resilient to fire events, with tree stands often returning quickly to a pre-fire state. Additionally, *E*. *regnans* cohorts are predominately even-aged, and require stand-replacing fire events for their persistence in the landscape [7]. In contrast, Tasmanian wet eucalpyt forests, located further south, are largely dominated by *Eucalyptus delegatensis* in the central and northern parts of the island [10]. While *E*. *delegatensis* is often also regarded as being an obligate seeder (e.g., [11]), the response of Tasmanian *E*. *delegatensis* forests to fire is relatively understudied. Additionally, it is unknown whether Tasmanian wet-sclerophyll forests, (the same broad ‘forest type’), respond to fire similarly to the tall eucalypt forests of Victoria. Such a question is critical to understanding how to conserve and manage these systems in the face of future fire threats.

In this study, we examine the influence of wildfire on the structure and function of Tasmanian wet eucalypt forests, and evaluate the implications of wildfire for biodiversity and sustainable forest management. More specifically, we aim to: 1) determine the mortality of eucalypt, acacia, understory tree species, and the tree fern *Dicksonia antarctica*, following a severe fire event, 2) quantify the density and pattern of seedling establishment of eucalypt and acacia species, post-fire, and 3) evaluate faunal responses to fire. This research focuses on an area affected by the recent 2016 Tasmanian wildfires, which fortuitously intersected with a pre-existing forest monitoring plot [12]. This study not only provides region-specific information on the response of Tasmanian wet-sclerophyll forests to fire, but also has broader relevance to the forest ecology community, by demonstrating the valuable opportunities that can arise from establishing and maintaining forest-monitoring networks. Such opportunities are rare, and will be important for determining ecosystem resilience in the face of intensifying disturbance regimes, which are expected to be among the most severe climate change impacts on forest ecosystems in the future [13, 14].

## Materials and methods

### Study plots

The AusPlots Forest Monitoring Network is a monitoring plot network comprised of 48, 1 hectare (100 × 100 m) permanent plots across the Australian continent, established between 2012 and 2015, and designed for long-term ecological research with future repeat measurements planned. Each plot is gridded into 25 × 20 m square subplots. Two Tasmanian study plots were included in the present study—a burnt site (“Mackenzie”) (-41°37’49.08”, 146°15’33.48”) and a comparable undisturbed site (“Mt Maurice”) (-41°18’40.68”, 147°, 32’17.87”). The comparison site was chosen based on similarities between pre-fire biotic and physical characteristics, including species composition, density of the dominant tree fern *D*. *antarctica*, understory coverage of grasses, ferns, shrubs, bare ground, rocks and coarse woody debris, elevation, slope, annual temperature and annual rainfall. This decision was supported by examination of a nonmetric multidimensional scaling (NMDS) ordination of the 14 existing Tasmanian plots (see below for details on ordination methods). The disturbed site, Mackenzie, was burnt during an extensive bushfire that swept through the region in January/February 2016. The fire was predominantly of moderate severity, but included distinct patches of low and high severity burns. The fire involved substantial ground burn with moderate canopy scorching. Trees generally showed scar heights between 2 and 5 m, though did exceed 5 m in localised areas of the plot. Mt Maurice was untouched by this fire, with the last large fire estimated to have occurred in 1934 [12].

### Tree mortality

Prior to the fire event, all live stems within the plot exceeding 10 cm in diameter were identified to species level, tagged with a permanent, unique identifier, and attributed a X-Y coordinate between 0 and 100 in relation to a georeferenced corner of the plot (0,0). Dead trees were measured as above and assigned with codes describing the physical mechanism for mortality. Tree mortality was then assessed in December-January 2017, nearly 1 year after the wildfire event. All uniquely identified trees were re-visited, and dead trees were assigned with codes describing the physical mechanism for mortality (e.g., tree fall, fire). Any eucalypt tree that had not re-sprouted after this recovery period following the fire was presumed dead. Detailed field protocols for plot establishment are outlined in the AusPlots Forest Monitoring Network Survey Protocols Manual, available in Wood et al. [12].

### *Dicksonia antarctica* spatial mapping

All *D*. *antarctica* (tree ferns) greater than 1.3 m in height were spatially mapped following the tree survey methodology outlined above. Height was determined as the length from the base of the fern to the apex of the trunk. The alive status of each tree fern was assessed through the presence of live mature fronds or sprouting fiddleheads [15]. Any *D*. *antarctica* that did not have re-sprouting fiddleheads after the fire were presumed dead. Scorching and the presence of seedlings on the trunks of each tree fern were also noted. All *D*. *antarctica* were surveyed between December and January 2016/2017.

### Fuel load and coarse woody debris

Fuels and coarse woody debris were evaluated before and after the fire event for each site. Categorical information was collected on the amount of grass, shrubs and bare ground ranked categorically from 0 (none) to 3 (many) for each subplot. The number of sticks, logs and coarse woody debris (<5 m in length, >20 cm in diameter) were also counted for each subplot.

### Seedling counts

Density of eucalypt and acacia seedlings were measured in December 2016, 11 months after the fire. At each site, seedling density was measured within 50 × 1 m^2^ quadrats. Quadrat locations were determined through stratified random sampling, with two quadrats established per subplot. Other tree seedlings were also counted to show overall seedling density and, where possible, identified to species level. Percentage cover of litter, live vegetation, moss and lichen were recorded for each quadrat, as well as the number of animal scats within each.

### Fire severity

The severity of the wildfire was assessed across each of the 25 subplots within the burnt site. Severity was evaluated categorically according to the Ausplots Forest Monitoring Network Survey Protocols Manual [12]. Categories were as follows: severity 3 (high)–tree scorching greater than 5 m in height, with or without crown damage. Severity 2 (moderate)–lack of understory and tree scorching up to 2 m. Severity 1 (low)–minor scorching restricted to logs and lower trunks. Fire-severity data was collected on the 19th of January 2017.

### Invertebrates

Litter and ground-dwelling invertebrates were collected using standard pitfall traps [16] between December and January 2016/2017. One trap was allocated to a random position within each of the 25 subplots of each site (see Skvarla and Larson [17] for sample size adequacy in temperate areas). Traps were placed at minimum one meter apart to both reduce trap-to-trap interference, and avoid depletion of invertebrate populations across the sampling period [18]. Pitfall traps were constructed using clear plastic cups 9 cm in diameter, containing 2–3 cm of a dilute propylene glycol mixture and covered with a 150 × 150 mm elevated roof. Pitfall cups were also covered by 4 cm chicken wire to prevent access by reptiles, mammals and birds. Traps were emptied at 2-week intervals throughout the trapping period (three repeat samples). In the laboratory, invertebrates were sorted into functional categories according to morphospecies [19], counted and weighed. Only invertebrates of body length > 1 mm were included, as invertebrates smaller than this are not well sampled using pitfall trapping methods [20]. Biomass for each group was recorded using wet weight of invertebrates [21].

### Diurnal birds

Fixed-radius point counts were used to determine species occurrence at each site [22, 23]. Repeated 10-minute point counts were done at four posts within the plot to encompass the survey area. Posts were located at X and Y coordinates (20,80), (80,80), (20,20), (80,20), relative to a georeferenced corner of the plot (0,0). All species of birds heard and seen within a 20 m radius of the posts during the survey were recorded. Species observed outside the 20 m radius were noted separately. Between point counts, there was a minimum of 1 minute rest after moving to a new location, to allow the birds to settle [22]. Any species observed before or after the survey period were recorded to catalogue bird diversity at the sites, but were not included in the analysis.

Sites were surveyed after the dawn chorus, and completed between 7am and 8am (0.5–2 hours after sunrise). All surveys were done between late November and early January, which is the breeding season for many bird species, and when summer migrants have arrived [24]. Each site was assessed by a single person (experienced with bird identification) to reduce any potential observer biases. Temperature, wind, precipitation and time were noted at the beginning of each point count. No surveys were done during rainfall events, if temperature was below 7°C or above 24°C, or if wind speed exceeded ~11 km/hour [22]. There were four independent spatial and temporal replicates per site.

### Statistical analyses

Between-site differences in tree mortality and seedling density, and environmental predictors of tree mortality and seedling density for the burnt site, were estimated using generalised linear modelling (GLM), fitted via maximum likelihood. Tree mortality was analysed at two levels (individual tree and subplot) according to the level at which environmental information was collected. Predictors of tree mortality included diameter at breast height (DBH) and tree species. Predictors for subplot-level mortality included fire severity and slope. Seedling density was evaluated at the quadrat level, with fire severity, distance to the nearest live parent tree (post-fire), and the percent cover of live vegetation used as predictors. An ordered logistic regression (multinomial distribution) was used to evaluate the influence of topography and pre-fire abundances of grasses, shrubs, coarse woody debris and tree stems on fire severity.

Binomial error distributions with logit-link functions were used for all models assessing tree mortality (individual and sub-plot level), where trees that had died between sampling periods were coded as 0, and those that remained alive were coded as 1. A negative binomial error distribution was used for modelling seedling density, after confirmation of overdispersion [25].

We used the Akaike information criterion value, adjusted for small sample sizes (AIC_*c*_), to evaluate the GLMs. For this we created an *a priori* set of models, and calculated the AIC_*c*_ weight for each model fit. We then calculated model averaged coefficients using models with delta AIC_*c*_ values ≤ 10 [26]. We favoured an information theoretic approach instead of p-values, because it allowed us to make inferences on the likelihood of hypotheses across a set of model structures, and avoided potential problems with model-selection uncertainty [26].

The spatial distribution of *D*. *antarctica* throughout the burnt plot was evaluated using spatial point pattern analysis. The hypothesis of complete spatial randomness (CSR) was evaluated using Ripley’s K [27], Monte Carlo significance tests were calculated from 499 simulations of the observed spatial pattern, with an envelope distance of 50 and a 0.5 increment distance for envelopes. Deviation from CSR was determined by the model fit (u rank) and both Clarke and Evans and Donnelly summary statistics. Visual examination of the Ripley’s K function plot aided pattern identification; if the observed point pattern fell above (clustering/aggregation) or below (regularity) the confidence bounds. The null model of CSR was rejected in this case, as *D*. *antarctica* exhibited aggregation at distances of 2–50 metres. Therefore, first- and second-order aggregation models (inhomogeneous Poisson and Thomas cluster process, respectively), were used to assess the nature of the underlying aggregation (see [27]). The same analyses were also used to evaluate the spatial distribution of unburnt *D*. *antarctica* throughout the plot. Univariate analyses were used instead of a multi-type point process aggregation model, with fire scar as a mark, due to the low sample size of unburnt *D*. *antarctica* (*n* = 18).

Site-level forest structure and composition was evaluated using NMDS ordination. This gave a graphical representation of the change in biotic and physical characteristics of the burnt site, compared with other wet-sclerophyll sites across Tasmania. Ordinations included 1) species composition, based on the number of stems per species, 2) species composition, as informed by total basal area (m^2^) per species, 3) biotic and abiotic characteristics, including the number of living stems, number of dead stems, number of species, elevation, annual temperature (°C), annual precipitation (mm), total basal area (m^2^), eucalypt basal area (m^2^) and non-eucalypt basal area (m^2^), and 4) as above with additional information on the density of *D*. *antarctica*, understory coverage of grasses, ferns, shrubs, bare ground, rocks and coarse woody debris and slope. Ordinations were done using Bray-Curtis (dis)similarity matrices on square-root-transformed values. The quality of the ordination was assessed through generating “stress” values, which indicate the level of fit between the real dissimilarities and the ordination distances. Stress values < 0.1 suggest good fit, <0.2 reasonable fit, and >0.2 non-fit [28]. The suitability of ordinations were also assessed by visual inspection of a Shephard Plot. This depicts the relation of ordination distances to actual measured distances, where good fit is indicated by a monotonic increase in points [29].

To evaluate the impact of fire on bird composition, we used a binomial test of equal ratios between the sites, with a 0.5 null expectation. Bird response variables were grouped into guilds that represented habitat use and feeding preference. Guilds used in the analysis included nectarivores, open-ground insectivores, understory insectivores (insectivores that occupy and forage within the low to mid canopy), canopy insectivores, omnivores, and granivores [30]. Response metrics were derived by summing the frequency of recordings of each species according to guild. This represented the number of opportunities (out of 16) at a given site that a species was detected, and encapsulated four independent spatial and temporal replicates per site. The influence of fire on invertebrate composition was also evaluated using a binomial test of equal ratios, analysed by the summed abundance per group.

## Results

### Fire severity

**Fig 1:**
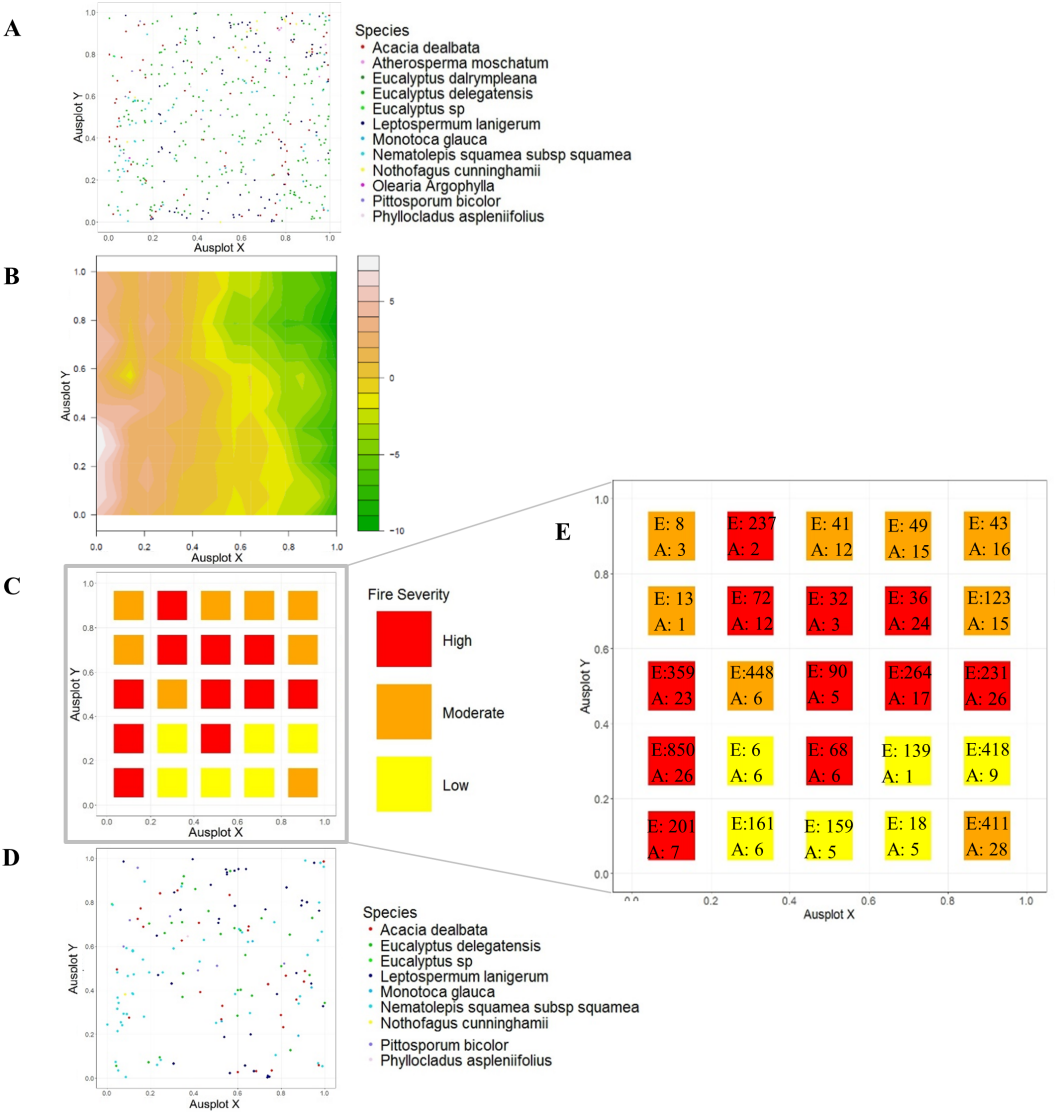
Live- and dead-tree distribution across the burnt plot. A) Distribution of live trees before the fire event. B) topographic map of the plot, with colours representing height gradient. C) Fire-severity rating per subplot, where High = Scorching >5 m height and/or crown damage, Moderate = Lack of understory and scorching up to 2 m, Low = Minor scorching on logs and lower trunk. D) Post fire distribution of dead trees. E) Enlarged fire severity map with number of eucalypt (E) and acacia (A) seedlings per subplot. The average number of eucalypt and acacia seedlings for each severity class were as follows- High: 111 and 7, Moderate: 71 and 6, Low: 75 and 3.

The fire burnt 84.1% (*n* = 403) of all trees in the Mackenzie plot, and 90.4% (*n* = 169) of all measured *D*. *antarctica*. Eleven subplots were burnt at a high severity, eight at moderate, and six at low severity (Fig 1C). No landscape variables explained a substantial proportion of the variation in fire severity, with the null model preferred (w_i_ = 0.29) (Table 1), and the highest deviation explained (%DE) being only 4.2% in the saturated model. Model-weighted variables had some predictive capacity for fire severity, as summarised in Table 1.

**Table 1:** Model-averaged parameter estimates of models.

Model and predictor variable	Estimate	(LCI, UCI)	Weighted SRC
Fire Severity			
High | low	0.35	(0.05, 0.84)	0.20
Low | med	0.59	(0.13, 0.93)	0.12
Slope	0.49	(0.46, 0.52)	0.17
CWD	0.40	(0.12, 0.77)	0.16
Grass	0.39	(0.08, 0.83)	0.06
Shrub	-	-	-
Trees	0.47	(0.18, 0.78)	0.30
Tree level mortality			
Intercept	0.18	(0.08, 0.36)	0.19
*Eucalyptus delegatensis*	0.80	(0.68, 0.89)	0.25
*Leptospermum lanigerum*	0.63	(0.45, 0.79)	0.09
*Nematolepis squamea*	0.15	(0.06, 0.36)	0.18
Diameter	0.51	(0.51, 0.52)	0.29
Subplot level mortality			
Intercept	0.40	(0.29, 0.52)	0.24
Low severity	0.34	(0.23, 0.48)	0.34
Moderate severity	0.37	(0.27, 0.49)	0.33
Slope	0.25	(0.01, 0.95)	0.08
Eucalypt seedlings			
Intercept	0.99	(0.98, 1.00)	0.78
Live vegetation	0.49	(0.48, 0.50)	0.13
Parent proximity	0.50	(0.45, 0.55)	0.01
Low severity	0.44	(0.21, 0.70)	0.02
Moderate severity	0.44	(0.23, 0.66)	0.02
Unburnt	0.35	(0.08, 0.76)	0.03
Acacia seedlings			
Intercept	0.93	(0.84, 0.97)	0.38
Parent proximity	0.47	(0.44, 0.49)	0.18
Low severity	0.35	(0.20, 0.53)	0.12
Moderate severity	0.46	(0.32, 0.61)	0.03
Unburnt	0.03	(0.00, 0.21)	0.23
Live vegetation	0.50	(0.50, 0.51)	0.05

The fire-severity model was fitted using ordinal regression, the tree-level and sub-plot level mortality using binomial GLMs, and seedling models using negative-binomial GLMs. Weighted SRC indicates the importance of each variable as the scaled z score, expressed as a weighted value. All coefficients have been converted from log odds to probability. See S1 Table for AICc and model-selection results. Note: sign of the estimate indicates a positive or negative effect on the dependent variable, CI overlap with zero indicates no statistically discernible effect. Non-fit of the parameter is demonstrated by a -.

### Mortality

A third of all trees died at the burnt site (33%, *n* = 159). This contrasts sharply with the unburnt site, where only 6 trees (1.4%) died over the survey period. The generalised linear model on individual tree mortality by site (binomial with logit link) showed no overlap in Confidence intervals: burnt: 0.67, CI: 0.63–0.71; unburnt: 0.97, CI: 0.93–0.99, %DE = 20.0.

Individual tree mortality within the burnt site was strongly influenced by both species and DBH (*w*_*i*_ = 1.00, %DE = 31.5). *E*. *delegatensis* showed the highest probability of surviving the fire (0.8). This was 5.3 times greater than *N*. *squamea*, 4.4 times greater than *A*. *dealbata* and 1.3 times greater than *L*. *lanigerum* (Table 1). DBH of individual trees has a small influence on the likelihood of survival at the burnt site, where a 10 cm increase in DBH secured a 0.1-fold increase in survival probability (Table 1).

Sub-plot tree mortality was also substantially lower at the unburnt site (burnt: 0.33, CIs: 0.29, 0.38; unburnt: 0.03, CIs: 0.01, 0.06), (%DE = 76.0). Mortality within the burnt site was marginally influenced by fire severity and slope of subplots (Table 1), though models explained little deviance (%DE = 0.7).

### Seedlings

A total of 4,477 eucalyptus seedlings were counted within 50 × 1 m^2^ quadrats at the burnt site, and 279 acacia seedlings. No eucalypt or acacia seedlings were counted at the unburnt site. *Tasmannia lanceolata*, *Phyllocladus aspleniifolius*, and *Nothofagus Cunninghamii* seedlings were found in quadrats at the unburnt site but not the burnt site. Despite variation in the fire severity across the burned plot (see above), we were unable to explain any substantial spatial variation in seedling density within sites (S1 Table). Acacia seedling density increased marginally with fire severity at the burnt site: quadrats that experienced high-severity burning had 1.2-fold more seedlings than moderately burnt quadrats, 2-fold more than the low intensity quadrats, and 35-fold more than in unburnt quadrats (see Table 1 for all models). Eucalypt seedlings showed a similar response, with seedling density only 1.3-fold higher when burnt at a high severity compared to moderate or low intensity burning. Eucalypt density was only 1.9-fold higher in high severity patches than in unburnt patches, however. Neither eucalypt nor acacia seedling growth was substantially affected by distance from parental trees, nor by live vegetation within plots (Table 1).

### Dicksonia antarctica

**Fig 2:**
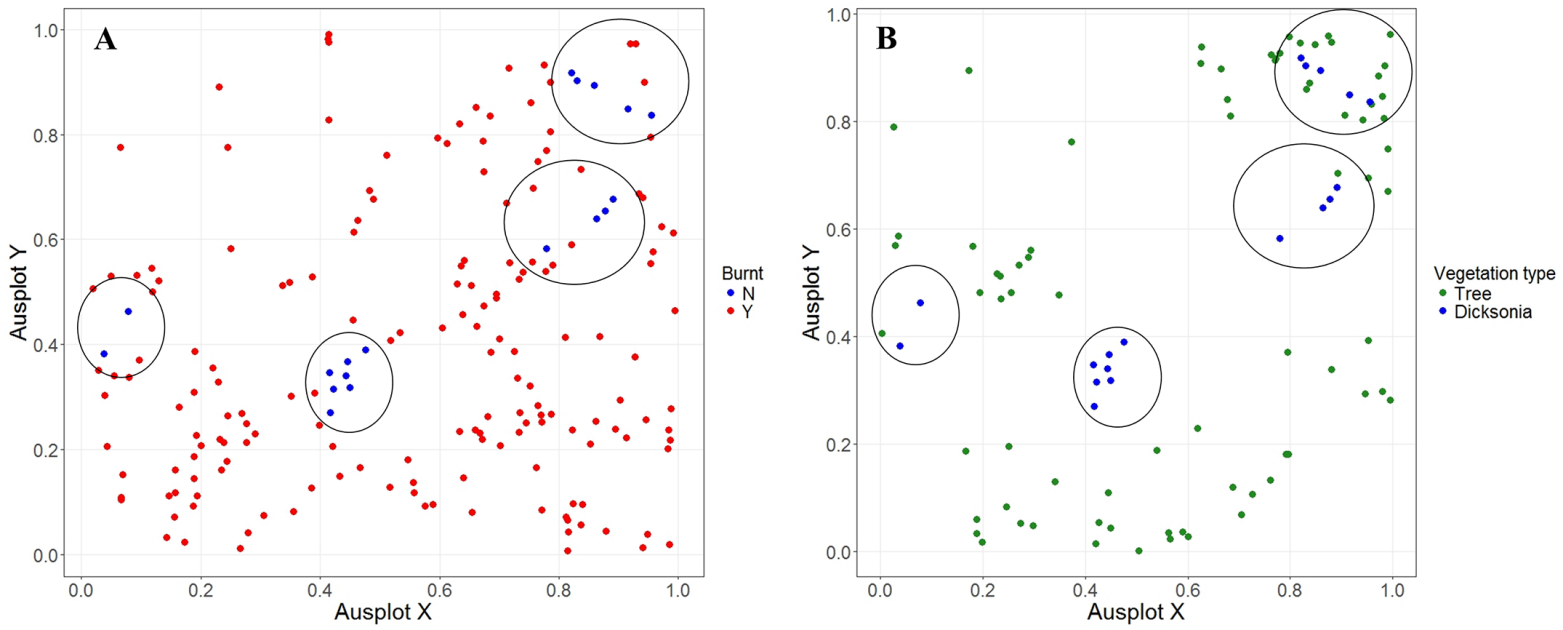
Spatial maps of *Dicksonia antarctica* and unburnt trees within the burnt site. A) Map of burnt and unburnt *D*. *antarctica* clustering at the burnt site, B) Cluster map of unburnt *D*. *antarctica* and unburnt trees. Axes represent increments of 20 m, with the total plot size equal to 100 x 100 m (1 hectare). Clusters of unburnt *D*. *antarctica* are highlighted with circles.

We observed significant deviation from complete spatial randomness within the univariate spatial analysis, with the plot of the enveloped Ripley’s K function suggesting an aggregated pattern (S1 Fig). *D*. *antarctica* fit well to a first-order aggregation null model, and poorly with a second-order null model (S1 Fig). This suggests environmental heterogeneity may be driving the pattern of *D*. *antarctica* within the burnt site, rather than species interactions. Unburned *D*. *antarctica* also showed evidence of spatial clustering at distances of 2 to 35 m (S1 Fig). The distributional plot of *D*. *antarctica* shows four separate clusters of unburnt *D*. *antarctica*, arranged roughly diagonally across the centre of the plot (Fig 2). This follows a small creek line which likely provided protection within or along the outer boundary of the stream. Indeed, the clustering of unburned *D*. *antarctica* broadly occurred in the same areas as the unburned trees (Fig 2), supporting the creek-line explanation of shelter from fire.

### Forest structure and composition

**Fig 3:**
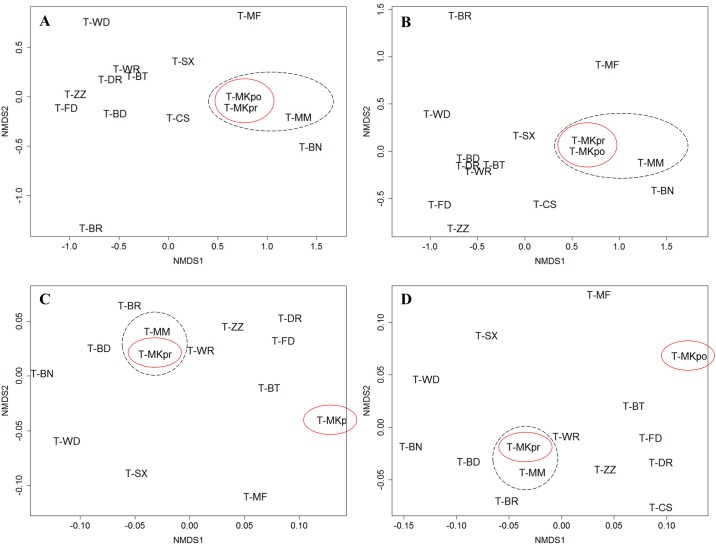
Graphical representation of the change in biotic and physical characteristics of the burnt site, compared with other wet sclerophyll sites across Tasmania. Ordinations include A) species composition based on tree stems, B) species composition based on basal area, C) other site characteristics with fuels and D) other site characteristics without fuels. The burnt plot (pre and post fire) is circled in red. The black circle encompasses the burnt plot in relation to the reference unburnt plot. Ordinations were conducted using Bray-Curtis (dis)similarity matrices on square root transformed values.

The pre-fire and post-fire site structure was differentiated in terms of general site characteristics: this varied depending on whether fuels were included in the ordination. With fuels the burnt site post fire structure was not similar to any other Tasmanian site (Fig 3C: stress = 1.08; Fig 3D: 1.66). By contrast, there was no obvious change in species composition before and after the fire, with both time points still similar to other Tasmanian sites (Fig 3A: stress = 1.04; Fig 3B: 1.08).

### Birds

A total of 33 different bird species were identified across both sites (Table 2). The frequency of bird detections differed by site, although low sample sizes limited statistical interpretation. Open-ground insectivores were more frequently detected at the burnt site (coef = 0.74, CI: 0.58–0.86, null expectation is 0.5), as were omnivores (coef = 0.63, CIs: 0.50–0.75). There were fewer honeyeaters at the burnt site than the unburnt site (coef = 0.29, CIs: 0.14–0.48). When nectarivores were analysed as a guild, with wattlebirds included, there was no statistical difference between the sites (coef = 0.48, CIs: 0.32–0.63, confidence intervals overlaps with 0.5). Understorey insectivores (coef = 0.43, CI = 0.33–0.54), canopy insectivores (coef = 0.41, CI = 0.32–0.51), and granivores (coef = 0.48, CI = 0.29–0.67) did not differ significantly between sites. See Table 2 for bird species within combined feeding and foraging guilds.

**Table 2:** Frequency of bird detections by site.

Species	Scientific name	Burnt	Un-burnt	Feeding guild	Foraging habitat	Analysis guild
Bassian Thrush	*Zoothera lunulata*	0	1	I	U	U-I
Thornbill	*Acanthiza sp*.	8	9	I	U	U-I
Tasmanian Scrubwren	*Sericornis humilis*	8	10	I	U	U-I
Golden Whistler	*Pachycephala pectoralis*	3	6	I	U	U-I
Olive Whistler	*Pachycephala olivacea*	4	11	I	U	U-I
Silvereye	*Zosterops lateralis*	6	6	I & N	U	U-I
Superb Fairy-wren	*Malurus cyaneus*	12	11	I	U	U-I
Dusky Robin	*Melanodryas vittata*	4	0	I	OG	OG-I
Flame Robin	*Petroica phoenicea*	13	9	I	OG	OG-I
Pink Robin	*Petroica rodinogaster*	8	1	I	OG	OG-I
Fan-tailed Cuckoo	*Cacomantis flabelliformis*	7	1	I	OG	OG-I
Dusky Woodswallow	*Artamus cyanopterus*	0	2	I & N	C	C-I
Eastern Spinebill	*Acanthorhynchus tenuirostris*	1	2	I & N	C	C-I
Grey Fantail	*Rhipidura albiscapa*	16	15	I	C	C-I
Satin Flycatcher	*Myiagra cyanoleuca*	1	12	I	C	C-I
Shining Bronze-Cuckoo	*Chrysococcyx lucidus*	10	10	I	C	C-I
Spotted Pardalote	*Pardalotus punctatus*	1	4	I	C	C-I
Striated Pardalote	*Pardalotus striatus*	14	16	I	C	C-I
Black-headed Honeyeater	*Melithreptus affinis*	3	4	N, F, I	C	N
Crescent Honeyeater	*Phylidonyris pyrrhopterus*	2	7	N, F, I	U	N
Strong-billed Honeyeater	*Melithreptus validirostris*	4	8	N, F, I	C & U	N
Yellow-throated Honeyeater	*Lichenostomus flavicollis*	0	3	N, F, I	C & U	N
Yellow Wattlebird	*Anthochaera paradoxa*	12	1	N, F, I	C & U	N
Black Bird	*Turdus merula*	0	1	O	G	O
Black Currawong	*Strepera fuliginosa*	15	5	O	G & C	O
Forest Raven	*Corvus tasmanicus*	5	0	O	G & C	O
Grey Shrike-thrush	*Colluricincla harmonica*	16	15	O	G	O
Laughing Kookaburra	*Dacelo novaeguineae*	2	0	O	G	O
Grey Currawong	*Strepera versicolor*	0	1	O	G	O
Yellow-tailed Black-Cockatoo	*Calyptorhynchus funereus*	1	3	G	U & C	G
Sulphur-crested Cockatoo	*Cacatua galerita*	1	0	G	U & C	G
Common Bronzewing	*Phaps chalcoptera*	1	0	G	G	G
Green Rosella	*Platycercus caledonicus*	11	12	G, N, I	U & C	G

Species frequency was calculated as the number of opportunities (out of 16) at which the species was detected, summed by guild. Abbreviations are as follows: Feeding guild: G = granivore, I = insectivore, F = frugivore, N = nectarivore, O = omnivore; Foraging habitat: C = canopy, G = ground, OG = open ground, U = understory; Analysis guild: C-I = canopy insectivore, OG-I = Open-ground insectivore, U-I = understory insectivore, G = granivore, N = nectarivore, O = omnivore.

### Invertebrates

We also detected differences in the abundances of invertebrates between the burnt and unburnt site, with varying directions of response dependant on the functional group (Table 3). There were significantly fewer mites and isopods at the burnt site compared to the unburnt site, while grubs, ants, millipedes and winged insects were more abundant. No differences where observed for abundance in spiders, beetles, caterpillars and snails. Interestingly, the total number of invertebrates were higher at the burnt site, but overall biomass was lower.

**Table 3:** Results from the invertebrate analysis.

Morphospecies	Burnt	Unburnt	Predicted response (as per [31])	ES(CIs)
Araneae (spiders)	117 (4.3)	125 (1.9)	+/-	0.48 (0.42, 0.55)
Acarina (mites)	10 (0.0)	55 (0.0)	-	0.15 (0.08, 0.26)
Grubs	524 (34.4)	59 (4.3)		0.90 (0.87, 0.92)
Isopoda	17 (0.1)	98 (0.3)	-	0.15 (0.09, 0.23)
Coleoptera (beetles)	249 (52.6)	239 (72.8)	+/-	0.51 (0.46, 0.56)
Hymenoptera (ants)	7 (0.1)	18 (1.0)	+	0.28 (0.12, 0.49)
Lepidoptera (caterpillars)	3 (0.3)	2 (11.5)		0.60 (0.15, 0.95)
Mollusc (snails)	9 (0.9)	5 (0.1)		0.64 (0.35, 0.87)
Myriapoda (Millipedes and centipedes)	87 (4.1)	32 (4.1)		0.73 (0.64, 0.81)
Winged insects	45 (0.7)	16 (0.3)	+	0.74 (0.61, 0.84)
Miscellaneous	(2.6)	(3.6)		-
Total	1074 (22.1909 g)	662 (44.3662 g)		0.62 (0.59, 0.64)

The abundance and proportional biomass (in brackets) for each invertebrate grouping are presented for the burnt and unburnt site, along with the effect size and confidence intervals from the sign test. The predicted response of each group are also summarised. In comparisons where the confidence intervals of the effect size overlaps with 0.5, there is no statistically detectable difference between sites.

## Discussion

This study, in presenting the results of a natural BACI experiment, is the first of its kind in Tasmania, and has allowed us to evaluate in detail the influence of fire on the structure and composition of cool-wet-sclerophyll forests. Despite moderate-to-high intensity burning across much of the exposed one hectare plot, the fire-driven mortality of eucalypt, acacia and tree ferns was low. Eucalypt and acacia seedling regeneration was also high in the burnt plot, but non-existent in the unburnt plot. These results contrast to responses of Victorian *E*. *regnans* and *E*. *delegatensis* forests to intense fire. These results will have implications for wet forest silvicultural practices in wet-sclerophyll forests, particularly under changing fire regimes.

### Immediate structural effects of fire

Disturbance by fire has long been recognised as a key driver of forest composition and structure [32]. A major mechanism leading to forest structural change is the mortality of individual large trees. In our fire-exposed study plot, mortality was predominantly observed in the characteristic ‘rainforest’ species. Substantial losses of these taxa were expected, given that rainforest trees are, by definition, highly pyrophobic [33]. These species have thin bark that offers little protection from heat damage [34]. Interestingly, *Nothofagus cunninghamii* (myrtle beech) appeared somewhat resistant to burning, with only 12.5% (*n* = 1) of the burnt trees being killed by the fire; this is consistent with observations of regeneration in this species after low-intensity burning [35]. Importantly, the spatially heterogeneous nature of the fire allowed the preservation of many rainforest species throughout the landscape as a whole. For instance, 19% (*n* = 11) of *N*. *squamea* stems were sheltered from the fire, creating a small refuge for this species. This patterning helped to preserve all rainforest species on this site, except for the sparsely distributed *Monotoca glauca* and *Phyllocladus aspleniifolius*, which were completely removed. With proportionally fewer rainforest understory species, the post-fire character of the tree community at the Mackenzie plot became less like that of other mature wet-sclerophyll forests in Tasmania, including the matched control (unburnt) site at Mt Maurice. This altered composition is likely to be reflective of an earlier successional stage for this forest type, by resetting the slow successional transition into rainforest.

In contrast, the eucalypt and acacia trees at the fire-exposed site had a high rate of survival, with a loss of only 12.6% (*n* = 28) of all burnt *E*. *delegatensis*. Mortality was relatively constant across the plot, regardless of local severity, and was predominantly observed in immature trees with small diameters. These survival rates are more favourable than those reported in other studies of equivalent (high) burn severity, although these often encompass considerable heterogeneity. A Victorian study by Benyon and Lane [36] showed mortality rates of *E*. *delegatensis* and *E*. *regnans* to be 25% in low-severity burns involving minimal-to-no crown scorch. Mortality after medium-intensity fire ranged from 61% to 96%, for “moderate crown scorch” and “crown scorch” severity groupings. Survival after high-severity crown burn was close to zero. Our observations are also considerably lower than mortality estimates used in recent Victorian bushfire response modelling (e.g., [37]). This suggests that Tasmanian *E*. *delegatensis*, particularly large mature trees, are potentially more fire tolerant than generally assumed [38]. In addition, 14% (*n* = 24) of the surviving, but burnt, *E*. *delegatensis* at Mackenzie showed aerial resprouting. This is low in comparison to other eucalypt species [36], but important to note given that this species is commonly cited as an obligate seeder (e.g., [39]).

### Future structural effects of fire

In addition to the direct mortality of trees caused by fire, the long-term composition and structure of canopy species will be shaped by understory composition. In wet-sclerophyll forests, *D*. *antarctica* play a significant but often understated role in influencing forest structure. This species of ‘man fern’ regularly survives fire, and is often the first to regenerate following burning [40]. Fast re-establishment of fronds will create sheltered environments, and thereby regulate the recovery of other regenerating species. *D*. *antarctica* trunks also provide a moist substrate to support the establishment of other seedlings, including epiphytes and tree species [41]. In our study 99% (*n* = 184) of burnt *D*. *antarctica* survived the fire, exhibiting some form of regeneration a year after the fire event. The ability of *D*. *antarctica* to survive bushfires has previously been attributed to the tree fern’s thick outer layer which protects the inner apex from damage. In addition, we suggest that the spatial patterning of *D*. *antarctica* can also influence its survival. Our spatial analysis revealed an aggregation pattern of unburnt ferns, with clustering between 2 and 50 m. This is likely to reflect an aggregation around water sources, which would allow the species to take advantage of burn heterogeneity, and may further increase their chance of surviving high-severity fires. This is important, as clustering of *D*. *antarctica* in discrete areas will influence forest structure by, for example, creating shading effects, and discouraging shade-intolerant species, like eucalypts, from establishing in these areas.

Fire also plays a critical role in shaping the future structure and composition of sclerophyllous forests via promotion of seedling germination and establishment. In this study eucalypt and acacia seedlings were only found in quadrats that had been exposed to fire, and none were observed in the unburnt control plot. The density of germinating eucalypt seedlings was particularly high, averaging 90 per m^2^ (c.f. acacia = 6). The stimulation of eucalypt seedling germination and establishment by fire is well known (e.g., [38]). Wet-sclerophyll forests, in particular, require fire events for the germination of eucalypt seedlings, and maintenance of eucalypt trees into the future. While it is generally thought that wet-sclerophyll forests dominated by *E*. *delegatensis* require high-intensity stand-replacing events to maintain the dominance of eucalypts in the landscape (e.g., [42]), this study suggests that this is not a universal rule, as eucalypts at our site showed substantial germination after a predominantly moderate-severity fire. Monitoring of seedling growth into the future would be valuable to quantify the number of eucalypts that survive into maturity and actually establish in the future canopy.

Interestingly, while seedling germination at a landscape scale was dependent on the occurrence of the fire event, there was no evidence of fine-scale patterning in density for either eucalypt or acacia seedlings. Previous studies have demonstrated the role of fire severity and proximity to parent trees on determining patterns of seedling establishment [39]. Although variability in the density of seedlings across quadrats was high (range 0–799 for eucalypts, and 0–23 for acacias), these predictors explained little of this variation. The lack of influence from parent trees may reflect the high density and relatively uniform spread of parent eucalypt and acacia trees throughout our plot, coupled with a high potential for seed dispersal in these species [43].

### Management implications

These results have implications for the assumed requirement of stand replacement in Tasmanian forests. As an “obligate seeder” and fire sensitive eucalypt, *E*. *delegatensis* is generally assumed to undergo stand replacement following wildfire events, and predominantly exist in even aged stands. In this region, however, the fire was far from a stand-replacing event. This is consistent with other forest-fire research in Tasmania, which have reported multi-aged tall wet-sclerophyll forest stands following wildfires. Hickey et al. [44], for example, reported that less than half of all burns observed in their study area between 1850 and 1999 resulted in pure regrowth stands. While stand-replacing fires can and do occur in Tasmania, complete stand replacement following wildfires, particularly in tall wet eucalypt forests, is potentially less common in Tasmania than generally believed. This contrasts with Victorian fire-response literature, where stand replacing events appear to be more common. This may relate to differing levels of fire severity experienced in each state due to climatic contrasts, coupled with differences in fire sensitivity between the two dominant eucalypt types (i.e. *E*. *regnans* in Victoria vs *E*. *delegatensis* in northern and central Tasmania). It is important to note that areas outside our immediate research plot did experience higher-severity burns, in some cases involving complete crown removal. The mortality of eucalypts in these areas was much higher, and included areas of complete stand death. However, the heterogeneous nature of fire retained multi-aged forest stands in the landscape as a whole.

This observation is particularly important for forest management practices, because stand-replacing fires underpin the design of clearfell, burn and sow (CBS) operations in *E*. *delegatensis* forests. During CBS, all merchantable trees are removed, and a new stand is initiated with a high-intensity regeneration burn and aerial sowing of eucalypt seed. This is often argued to be positive for forest regeneration and biodiversity because it mimics the frequency and scale of natural stand-replacing fires [7]. Tasmanian *E*. *delegatensis* forests are currently managed under both even-aged (CBS) and uneven-aged (partial harvest) silvicultural systems [45]. The application of CBS in Tasmania, however, may be inappropriate if stand-replacing fires are relatively uncommon, and are unnecessary for the maintenance of *E*. *delegatensis* in the landscape, as observed in this study. Inappropriate assumptions about regeneration and resilience of these systems might have negative effects on biodiversity, both in terms of understory plant composition and faunal diversity [9, 46].

### Implications for faunal diversity

Changes to the community structure of forest plants will invariably also impact existing faunal communities. We found some guild-specific avifaunal responses to the fire event; in particular, the open-ground insectivores (robins and cuckoos) were observed more often at the burnt site, whereas honeyeaters were more common at the unburnt control site. There were only marginal differences for the other feeding guilds. Victorian-based studies have documented similar avian responses, with persistence in the landscape dependant on resource availability after the fire.

The abundances of ground-dwelling invertebrates also differed between sites. There were significantly fewer mites and isopods at the burnt site, while winged insects, grubs, ants and millipedes were more abundant. These responses are similar to previously documented invertebrate responses in dry sclerophyll forests [31]. Flightless, ground-dwelling invertebrates are likely to have been killed in the fire, and have low mobility for re-colonisation, contributing to their low abundances at the burnt site. In contrast, winged insects have more potential to migrate into disturbed areas in response to rejuvenating foliage. Further, we detected no differences in the abundances of spiders, beetles, caterpillars and snails. The responses of beetles and spiders are likely to be influenced by the feeding and functional traits of invertebrates, which were not distinguished in this study. For instance, predatory ground beetles are more likely to increase following fire, in comparison to herbivorous beetles, following reduced cover for prey [47]. Similarly, while spiders in general are thought to be sensitive to fire (e.g., [31]), non-web-building, hunting spiders are more likely to be favoured by open habitat [47]. Interestingly, the total numbers of invertebrates were higher at the burnt site, but overall biomass was lower, suggesting a potential change in the size structure of invertebrate communities in fire disturbed areas. While our inferences are constrained to coarse taxonomic group, these findings contribute to the currently sparse knowledge of invertebrate responses to natural disturbances in non-conifer forest types, and serve as a basis for comparison into the future.

Overall, this study demonstrates the opportunities that can arise from establishment of forest-monitoring plots. Such plots can provide insight, not only into unpredictable disturbance events, but also for the understanding of long-term functioning and pattern–process interactions within ecosystems (when monitored repeatedly), which are central questions within forest ecology (e.g., [12, 13]). Importantly, these types of studies also reflect a balance between the scale and quality of information collected. At the landscape scale, this study covered only two 1-hectare research plots, though the size of these plots enabled us to capture the natural variation and spatial patterns of the landscape. As a result, we were able to evaluate questions at different scales (e.g. landscape: *n* = 2, subplot: *n* = 25, quadrat: *n* = 50, or individual tree: *n* = 480) depending on the nature of the question asked. New monitoring plots should aim to be at least one hectare in size, or alternatively, aggregates of smaller plots in a given landscape should provide sufficient coverage to adequately capture landscape variation, and thus maximise their value into the future.

## Conclusion

Here we report on the results of a natural BACI forest experiment, taking advantage of pre-existing monitoring plots established by the AusPlots forest monitoring network [12] and a mensurative experimental treatment provided by a natural disturbance event. This study is the first of its kind in Tasmania, permitting us to evaluate the influence of fire on the structure and composition of cool-wet-sclerophyll forests, in terms of both the immediate and potential future effects of burning, as well as associated faunal responses. We found that, despite moderate-to-high intensity burning in patches within the exposed plot, the mortality rates of eucalypt, acacia and fern were low. Impacts were higher for fire-sensitive rainforest species, though this group was able to persist in unburnt refugial areas, enabling their preservation in the landscape. The regeneration of eucalypt and acacia seedlings was high in the burnt plot, but non-existent in the unburnt control. These results contrast to studies from mainland Australia (Victoria), which showed much higher mortality rates for *E*. *delegatensis*, and seedling emergence restricted to high intensity fires. This suggests that Tasmanian wet-sclerophyll forests respond to fire differently to their more northerly counterparts, despite being classed as the same forest type. This has implications for wet-forest silvicultural practices in Tasmania, and highlights the need to consider Tasmanian and Victorian eucalypt forests separately when modelling responses to global change, particularly in the face of changing fire regimes. We also found some group-specific avifaunal and invertebrate responses to the fire event, which were broadly reflective of responses document in other Victorian-based studies. This suggests fire-driven floristic changes can impact faunal communities similarly between these forest communities. The establishment of forest monitoring plots, which provide detailed baseline information and are designed for repeated re-measurements over decades, are critical for providing such opportunities to take advantage of natural experiments, and for determining ecosystem resilience in the face of intensifying disturbance regimes imposed by ongoing climate change.

## Supporting information

S1 Table Model selection results (burnt site only). AICc = Akaike’s information criterion corrected for small sample sizes, wi = Akaike weight, DE = deviance explained by the fitted model. Bolded results indicate the models included in model averaging for parameter estimates (ΔAICc <10). Symbols represent the family of the test: *binomial, φ Negative binomial.
(DOCX)

S2 Table Summary statistics for the subplot mortality analysis. Variables included the number of trees alive and dead for both the pre- and post- fire surveys, and the severity of the fire in each subplot. Severity categories were as follows: severity 3 (high)–tree scorching greater than 5 m in height, with or without crown damage. Severity 2 (moderate)–lack of understory and tree scorching up to 2 m. Severity 1 (low)–minor scorching restricted to logs and lower trunks. The burnt site (“Mackenzie”) is coded as T-MK, and the unburnt site (“Mt Maurice”) as T-MM.
(DOCX)

S1 Fig Results of the Dicksonia antarctica spatial point pattern analysis (SPPA). Null models of each panel are as follows: A) Univariate complete spatial randomness, all *D*. *antarctica*, B) Univariate first order aggregation, all *D*. *antarctica*, C) Univariate second order, all *D*. *antarctica*, D) Univariate complete spatial randomness, unburnt *D*. *antarctica*, E) Univariate second order, unburnt *D*. *antarctica*. Panels show plots of the enveloped Ripley’s K function. Grey shading depicts 95% confidence intervals, and the solid black line is the fitted model. Non-fit to a model at a given distance (r) is indicated where the fitted line falls outside the confidence intervals. R represents the Donnelly test statistic, where R > 1 suggests ordering, and R < 1 clustering, and u is the u-rank for model fit. For all panels, the distances (in metres) of clustering is indicated by the x axis.
(DOCX)

S1 Dataset All floristic and faunal post-fire data, collected between November 2016 and January 2017. Spreadsheet includes data on: Seedling counts, fire severity, *Dicksonia antarctica* spatial locations, tree mortality, bird frequencies and invertebrate abundance and biomass. Explanations of data are contained within the spreadsheet.
(XLSX)
