# Supplementary material for: Impact of intense disturbance on the structure and composition of wet-eucalypt forests: A case study from the Tasmanian 2016 wildfires
Source: PLoS One. 2018 Jul 20;13(7):e0200905. doi: 10.1371/journal.pone.0200905 (PMC6054383; doi:10.1371/journal.pone.0200905)
Supplement: S1 Table — AICc = Akaike’s information criterion corrected for small sample sizes, wi = Akaike weight, DE = deviance explained by the fitted model. Bolded results indicate the models included in model averaging for parameter estimates (ΔAICc <10). Symbols represent the family of the test: *binomial, φ Negative binomial. (DOCX) [file pone.0200905.s001.docx]

**S1 Table. Model selection results (burnt site only).** AIC*_c_*= Akaike’s information criterion corrected for small sample sizes, w*_i_*= Akaike weight, DE= deviance explained by the fitted model. Bolded results indicate the models included in model averaging for parameter estimates (ΔAIC*_c_* <10). Symbols represent the family of the test: *binomial, φ Negative binomial.

| **Response variable and model** | **df** | **AIC***_c_* | **ΔAIC***_c_* | ***w_i_*** | **DE** |
| --- | --- | --- | --- | --- | --- |
| Tree level mortality ^*^ |  |  |  |  |  |
| Diameter + species | **5** | **392.1** | **0.0** | **1.00** | **0.32** |
| Species | 4 | 422.5 | 30.4 | 0.00 | 0.26 |
| Diameter | 2 | 443.0 | 50.9 | 0.00 | 0.21 |
| Null | 1 | 559.8 | 167.7 | 0.00 | 0.00 |
| Subplot level mortality ^*^ |  |  |  |  |  |
| Severity | **3** | **118.5** | **0.0** | **0.66** | **0.07** |
| Severity + slope | **4** | **120.9** | **2.4** | **0.20** | **0.08** |
| Null | **1** | **122.1** | **3.7** | **0.11** | **0.00** |
| Slope | **2** | **124.5** | **6.0** | **0.03** | **0.00** |
| Eucalypt seedlings ^φ^ |  |  |  |  |  |
| Live veg | **3** | **539.6** | **0.0** | **0.55** | **0.01** |
| Null | **2** | **541.7** | **2.2** | **0.19** | **0.00** |
| Parent proximity + live veg | **4** | **541.9** | **2.4** | **0.17** | **0.01** |
| Parent proximity | **3** | **544.0** | **4.0** | **0.06** | **0.00** |
| Severity | **5** | **546.8** | **7.3** | **0.01** | **0.00** |
| Live veg + severity | **6** | **546.9** | **7.3** | **0.01** | **0.01** |
| Parent proximity + severity | **6** | **549.4** | **9.8** | **0.00** | **0.00** |
| Parent proximity + live veg + severity | 7 | 549.6 | 10.0 | 0.00 | 0.01 |
| Acacia seedlings ^φ^ |  |  |  |  |  |
| Parent proximity + severity | **6** | **274.1** | **0.0** | **0.59** | **0.07** |
| Parent proximity + live veg + severity | **7** | **276.1** | **2.1** | **0.21** | **0.07** |
| Severity | **5** | **277.0** | **2.9** | **0.14** | **0.05** |
| Live veg + severity | **6** | **279.3** | **5.3** | **0.04** | **0.05** |
| Parent proximity | **3** | **283.4** | **9.3** | **0.01** | **0.01** |
| Null | 2 | 284.6 | 10.6 | 0.00 | 0.00 |
| Parent proximity + live veg | 4 | 285.6 | 11.5 | 0.00 | 0.01 |
| Live veg | 3 | 286.5 | 12.5 | 0.00 | 0.00 |
