# Supplementary material for: Impact of intense disturbance on the structure and composition of wet-eucalypt forests: A case study from the Tasmanian 2016 wildfires
Source: PLoS One. 2018 Jul 20;13(7):e0200905. doi: 10.1371/journal.pone.0200905 (PMC6054383; doi:10.1371/journal.pone.0200905)
Supplement: S2 Table — Variables included the number of trees alive and dead for both the pre- and post- fire surveys, and the severity of the fire in each subplot. Severity categories were as follows: severity 3 (high)–tree scorching greater than 5 m in height, with or without crown damage. Severity 2 (moderate)–lack of understory and tree scorching up to 2 m. Severity 1 (low)–minor scorching restricted to logs and lower trunks. The burnt site (“Mackenzie”) is coded as T-MK, and the unburnt site (“Mt Maurice”) as T-MM. (DOCX) [file pone.0200905.s002.docx]

**S2_Table. Summary statistics for the subplot mortality analysis.** Variables included the number of trees alive and dead for both the pre- and post- fire surveys, and the severity of the fire in each subplot. Severity categories were as follows: severity 3 (high) – tree scorching greater than 5 m in height, with or without crown damage. Severity 2 (moderate) – lack of understory and tree scorching up to 2 m. Severity 1 (low) – minor scorching restricted to logs and lower trunks. The burnt site (“Mackenzie”) is coded as T-MK, and the unburnt site (“Mt Maurice”) as T-MM.

| **Variable** | **Mean** | **SE** | **Median** | **Min** | **Max** |
| --- | --- | --- | --- | --- | --- |
| T-MK |  |  |  |  |  |
| Alive_pre-fire | 19.20 | 6.56 | 19 | 8 | 33 |
| Dead_pre-fire | 5.16 | 2.52 | 6 | 0 | 9 |
| Alive_post-fire | 12.80 | 5.51 | 11 | 5 | 25 |
| Dead_post-fire | 11.52 | 4.61 | 11 | 2 | 20 |
| Died_in_fire | 6.36 | 3.06 | 7 | 1 | 14 |
| Severity | 2.20 | 0.80 | 2 | 1 | 3 |
| T-MM |  |  |  |  |  |
| Alive_pre-fire | 17.20 | 6.44 | 17 | 9 | 37 |
| Dead_pre-fire | 4.68 | 2.07 | 5 | 1 | 9 |
| Alive_post-fire | 16.96 | 6.41 | 16 | 9 | 36 |
| Dead_post-fire | 4.92 | 2.10 | 5 | 1 | 9 |
| Died_in_fire | 0.24 | 0.51 | 0 | 0 | 2 |
| Severity | NA | NA | NA | NA | NA |
