# Supplementary material for: Impact of intense disturbance on the structure and composition of wet-eucalypt forests: A case study from the Tasmanian 2016 wildfires
Source: PLoS One. 2018 Jul 20;13(7):e0200905. doi: 10.1371/journal.pone.0200905 (PMC6054383; doi:10.1371/journal.pone.0200905)
Supplement: S1 Fig — Null models of each panel are as follows: A) Univariate complete spatial randomness, all D. antarctica, B) Univariate first order aggregation, all D. antarctica, C) Univariate second order, all D. antarctica, D) Univariate complete spatial randomness, unburnt D. antarctica, E) Univariate second order, unburnt D. antarctica. Panels show plots of the enveloped Ripley’s K function. Grey shading depicts 95% confidence intervals, and the solid black line is the fitted model. Non-fit to a model at a given distance (r) is indicated where the fitted line falls outside the confidence intervals. R represents the Donnelly test statistic, where R > 1 suggests ordering, and R < 1 clustering, and u is the u-rank for model fit. For all panels, the distances (in metres) of clustering is indicated by the x axis. (DOCX) [file pone.0200905.s003.docx]

**S1 Fig. Results of the Dicksonia spatial point pattern analysis (SPPA).** Null models of each panel are as follows: A) Univariate complete spatial randomness, all Dicksonia, B) Univariate first order aggregation, all Dicksonia, C) Univariate second order, all Dicksonia, D) Univariate complete spatial randomness, unburnt Dicksonia, E) Univariate second order, unburnt Dicksonia. Panels show plots of the enveloped Ripley’s K function. Grey shading depicts 95% confidence intervals, and the solid black line is the fitted model. Non-fit to a model at a given distance (r) is indicated where the fitted line falls outside the confidence intervals. R represents the Donnelly test statistic, where R > 1 suggests ordering, and R < 1 clustering, and u is the u-rank for model fit. For all panels, the distances (in metres) of clustering is indicated by the x axis.


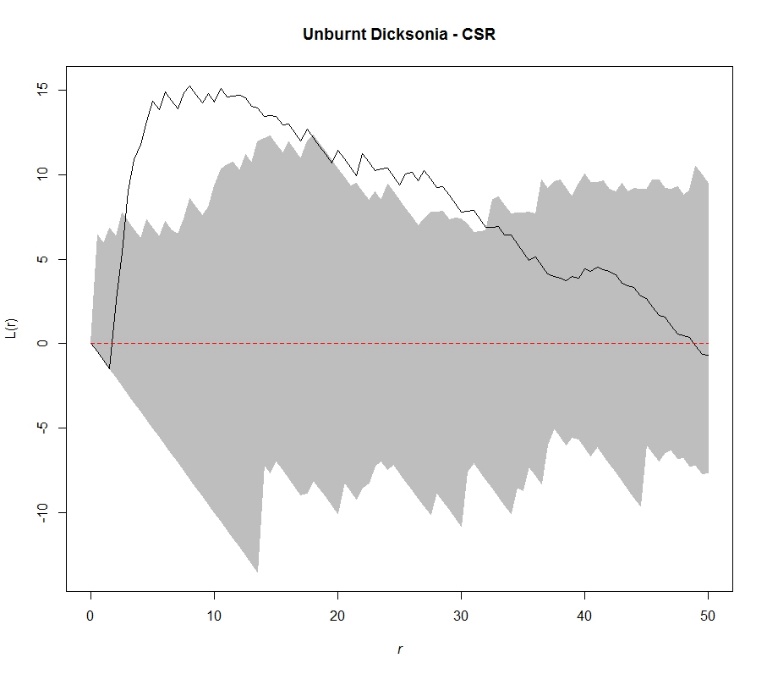

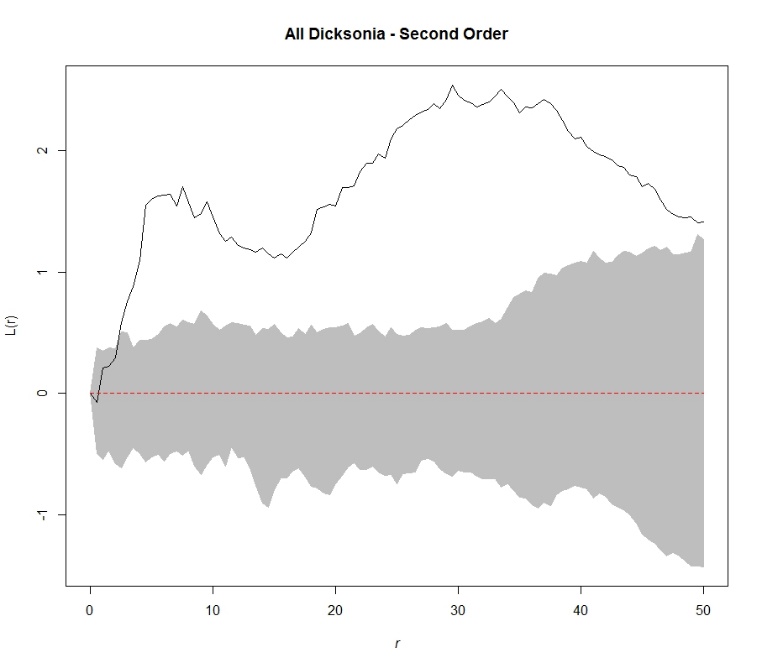

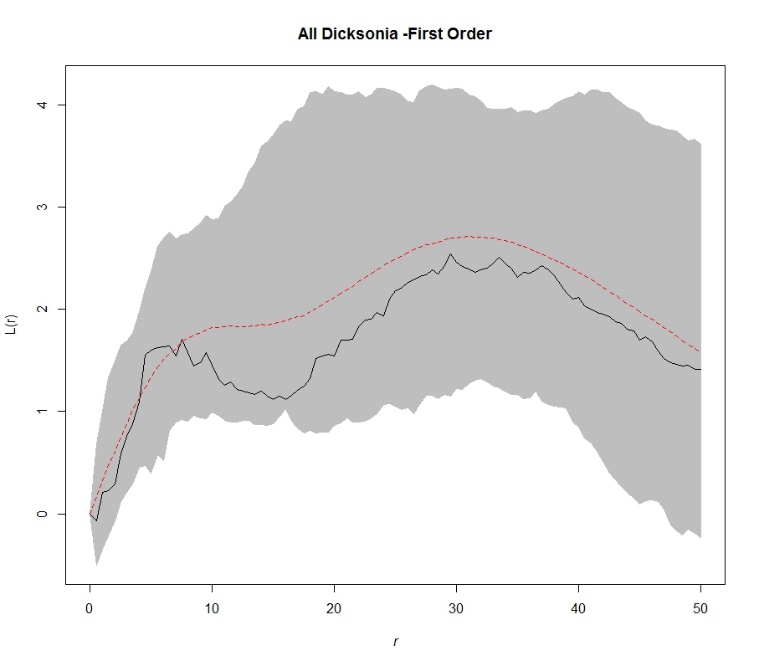

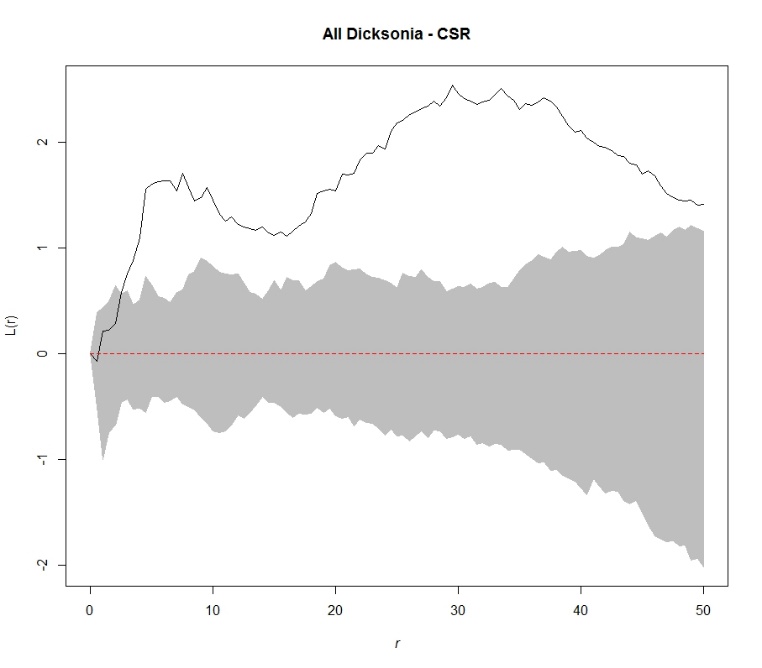


A

B

C

D

(R= 0.86, u= 2940.6, p=0.003)

(u= 119.76, p=0.46)

(u= 2940.6, p=0.01)

(R= 0.30, u= 1274600, p=0.01)


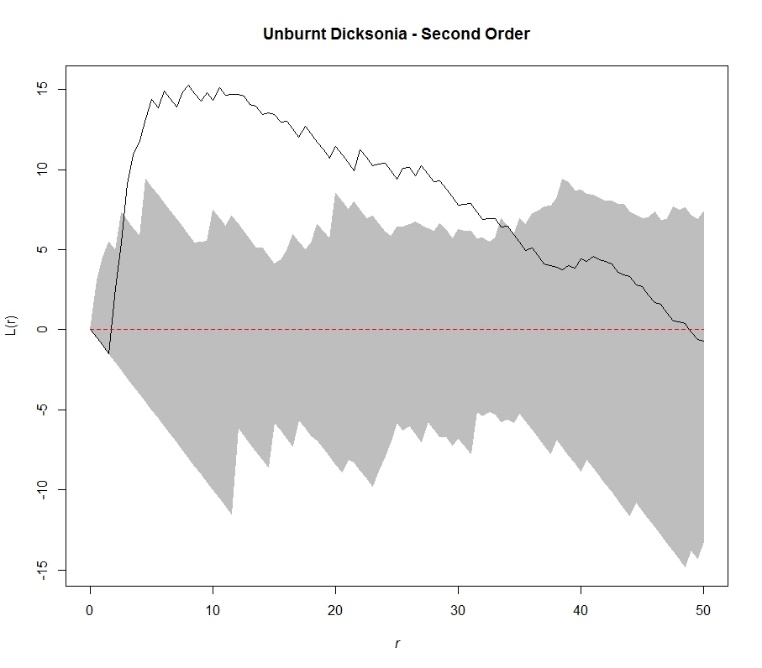


(u= 1274600, p=0.01)

E
